# Mediating role of body‐related shame and guilt in the relationship between weight perceptions and lifestyle behaviours

**DOI:** 10.1002/osp4.415

**Published:** 2020-03-25

**Authors:** K. M. Lucibello, C. M. Sabiston, E. K. O'Loughlin, J. L. O'Loughlin

**Affiliations:** ^1^ Department of Exercise Sciences, Faculty of Kinesiology and Physical Education University of Toronto Toronto Ontario Canada; ^2^ Carrefour de l'innovation et de l'évaluation en santé University of Montreal Hospital Research Center (CRCHUM) Montréal Québec Canada; ^3^ INDI Department Concordia University Montréal Québec Canada

**Keywords:** physical activity, sedentary behaviour, self‐conscious emotions, weight perception

## Abstract

**Introduction:**

A substantial proportion of individuals with overweight or obesity perceive themselves as ‘too heavy’ relative to ‘about right’. Perceiving one's weight as ‘too heavy’ is associated with lower levels of physical activity and higher levels of sedentary behaviour. However, the mechanisms underpinning the associations between weight perception and lifestyle behaviours have not been identified. Based on theoretical tenets and empirical evidence, the self‐conscious emotions of shame and guilt may mediate these associations.

**Methods:**

Participants were young adults (*n* = 618, M_age_ = 24.0 ± .6 years) who provided data on weight, weight perception, body‐related shame and guilt, physical activity and screen time.

**Results:**

Mediation analyses using the PROCESS macro indicated that shame and guilt significantly mediated the relationships between weight perception and physical activity and shame significantly mediated the relationship between weight perception and screen time.

**Conclusions:**

These findings provide preliminary evidence that self‐conscious emotions may be mechanisms by which weight perception influences physical activity and sedentary behaviour in young adults. However, longitudinal investigations of this mechanism are needed.

## 1 INTRODUCTION

High levels of physical inactivity and sedentary behaviour such as screen time are common in young adults.^1^, ^2^ Although longitudinal studies indicate that reduced physical activity engagement can result in weight gain over time,^3^, ^4^ the association between lifestyle behaviours such as physical activity or sedentary behaviour and weight status is not fully understood. Some cross‐sectional studies suggest that individuals with overweight or obesity engage in less physical activity and more sedentary behaviour than their normal weight counterparts.^5^, ^6^ Others report no differences in physical activity, sport participation^7^, ^8^ or sedentary time^9^ among adolescents and young adults with normal weight, overweight or obesity. These inconsistent results underscore the need for investigation into factors that contribute to the associations among weight status, physical activity and sedentary behaviour.

Body image is a multifaceted construct representing how one perceives, thinks, feels and acts towards the body.^10^ Specifically, perceptual body image^11^ may be important in better understanding the relationships among weight status, physical activity and sedentary behaviour. Weight perception refers to the subjective evaluation of weight status as underweight/too light, normal weight/just about right or overweight/too heavy.^12^ Although the descriptors used to label these perceptions differ across studies, the intent is to distinguish individuals who perceive themselves as an appropriate weight, as thinner or as heavier than they should or want to be. An inaccurate weight perception occurs when perceived weight is an overunderestimate or underestimate relative to actual weight status.

Previous investigations of inaccurate weight perceptions predominantly investigated individuals with a normal weight status who perceived themselves as overweight. This misperception was more common in females (25%–41%) than in males (5%–10%)^13^, ^14^, ^15^ and was associated with healthy (e.g., regular exercise) and unhealthy (e.g., fasting, vomiting and diet pills) weight control behaviours.^16^, ^17^ More recent evidence suggests that the probability of males and females perceiving themselves as overweight has declined over time.^18^ Additionally, the body image experiences of individuals with overweight or obesity are not homogenous,^19^ evidenced by weight underestimations across the lifespan.^20^, ^21^, ^22^ Specifically in young adults, approximately 50% of males and 20% of females with overweight or obesity do not perceive themselves as too heavy.^23^ This shift has been partially attributed to the increase in obesity prevalence, which is thought to have expanded body weight norms.^24^ Specifically, there appears to have been a recalibration of certain weights that people once perceived as overweight or obese that now fall within a ‘normal’ or ‘about right’ range.^24^

There are conflicting opinions regarding the implications of weight underestimation. Some argue that it represents progress in body acceptance and positivity.^25^ Others suggest that weight underestimation limits uptake of weight control behaviours.^26^, ^27^ Indeed, weight perceptions are associated with physical activity and sedentary behaviours after taking weight status into account.^28^, ^29^ Although the perception of being overweight is associated with weight loss intentions in males and females,^30^, ^31^ this perception (regardless of accuracy) is associated with *less* physical activity, sport and exercise,^28^, ^30^, ^32^ and more sedentary behaviours such as screen time^29^ than perceiving weight as about right. Overall, the literature suggests that perceptions of being too heavy increase the desire to lose weight but do not necessarily translate into adopting physical activity and nonsedentary behaviours. However, the decline in physical activity between adolescence and young adulthood^33^ limits the generalizability of these associations noted in adolescents to young adults.

Few studies have addressed the mechanisms underpinning the relationships among weight perceptions, physical activity and sedentary behaviour, although some suggest that body‐related self‐conscious emotions may partially explain these associations.^34^, ^35^ Self‐conscious emotions felt towards the body arise from self‐evaluative judgment^36^ and help motivate and regulate thoughts, feelings and behaviours.^37^ The intense self‐conscious emotion of *shame* occurs as a consequence of not living up to an internalized personal or social standard, which is attributed to a global flaw of the self.^38^ Shame is associated with avoidant behaviours in situations that evoke the emotion (e.g., those who are ashamed of their body avoid physical activity if the body is on display).^36^, ^39^ The self‐conscious emotion of *guilt* is described as feelings of tension, remorse and regret due to an action or behaviour over which the individual had control.^38^ Fixation on a transgression has been theorized to lead to behaviours that ‘fix’ the transgression (e.g., those who feel guilty about their body and weight control efforts will engage in physical activity to repair the ‘damage’ done by their behaviour).^36^, ^39^ Whereas some cross‐sectional evidence supports this association,^40^ other evidence has found no direct association,^39^, ^41^ or opposite patterns similar to shame.^42^ Based on these operationalizations and evidence, shame may lead to decreases in physical activity and increases in sedentary behaviour, whereas the associations among guilt, physical activity and sedentary behaviour require further investigation.

To date, researchers interested in the association between weight and body‐related self‐conscious emotions have focused on weight status.^34^, ^35^ Individuals who have overweight or obesity experience higher levels of shame and guilt, which is hypothesized to manifest due to experiences of weight stigma, and from recognizing that the body deviates from normative ideals. However, this tenet does not consider the role of subjective perception. Perceiving oneself as too heavy, as opposed to about right, can be conceptualized as evaluating oneself as discrepant from some internalized standard of an acceptable or ideal weight.^36^ Therefore, investigation into how weight perceptions evoke shame and guilt, and whether they contribute to avoidance or engagement in physical activity and sedentary behaviour, warrants empirical attention.

The purpose of this study is to investigate the associations among weight perceptions, physical activity and the sedentary behaviour of screen time, with self‐conscious emotions as mediators of the associations. Regardless of weight status, individuals who perceive themselves as too heavy were hypothesized to be engaging in less physical activity and more screen time than those who perceive their weight as about right. Shame and guilt were hypothesized to mediate these associations. Specifically, perceiving oneself as too heavy is associated with higher levels of shame and guilt; a higher level of shame is associated with less physical activity and more screen time; and a higher level of guilt is associated with more physical activity and less screen time.

## 2 MATERIALS AND METHODS

### 2.1 Participants

Data were drawn (*n* = 618) from the 2011–2012 cycle (the 22nd data collection cycle) of the Nicotine Dependence in Teens study.^43^ Seventh‐grade students (*n* = 1294, M_age_ = 12.8 years) in 10 schools in or near Montreal, Canada, were recruited for a longitudinal study beginning in 1999. Written parental consent was obtained at baseline, and participants provided consent in post high school data collections when they were of legal age. Schools differed in language of instruction (English and French), location (urban, suburban and rural) and a school socio‐economic indicator (low, moderate and high). Participants in the analytic sample were age 24.0 years on average (SD = 0.6, range 22–26); 58% female; and body mass index (BMI) was normal weight on average (24.0 kg/m^2^; SD = 4.1, range 18.6–38.9 kg/m^2^). Most participants identified their racial or cultural background as Caucasian (*n* = 513, 83%) and their marital status as single (*n* = 436, 70.6%). Study procedures were approved by the Montreal Department of Public Health Ethics Review Committee, the McGill University Faculty of Medicine Institutional Review Board, the Ethics Research Committee of the Centre de Recherche du Centre Hospitalier de l'Université de Montréal and the University of Toronto Research Ethics Board.

### 2.2 Measures

#### 2.2.1 Weight status and weight perception

BMI (kg/m^2^) was calculated from technician‐measured height and weight and then categorized based on the World Health Organization's (WHO's) ranges for underweight (<18.50 kg/m^2^), normal weight (18.50–24.99 kg/m^2^) and overweight or obese (≥25.00 kg/m^2^). Subjective weight perception was measured with a single self‐rated item on whether participants perceived their weight as ‘too thin’, ‘just about right’, ‘a little too heavy’ or ‘much too heavy’. Individuals who indicated ‘much too heavy’ (*n* = 32) were collapsed with ‘a little too heavy’ (*n* = 211) to create ‘too heavy’.^28^, ^31^

#### 2.2.2 Shame and guilt

Shame and guilt were assessed using the 12‐item Weight‐ and Body‐related Shame and Guilt scale (WEB‐SG).^34^ Participants reported shame in six items (e.g., ‘When I am in a situation where others can see my body [e.g. pool, changing room], I feel ashamed’) and guilt related to their body and weight control behaviours in six items (e.g., ‘When I can't manage to work out physically, I feel guilty’) on a Likert scale from 0 (never) to 4 (always). The average score was calculated for each subscale. The shame and guilt subscales have both demonstrated high internal consistency with Cronbach's alpha coefficients (*α*) of .92 and .87, respectively.^34^ The coefficients were *α*_shame_ = .89 and *α*_guilt_ = .90 in the Nicotine Dependence in Teens database.

#### 2.2.3 Physical activity

The short form of the International Physical Activity Questionnaire^44^ was used to assess levels of moderate‐to‐vigorous physical activity. Participants reported the number of days and average number of minutes per day that they engaged in each exercise intensity over the past week. The recommended truncation protocol (recode daily moderate and vigorous times exceeding 3 h to equal 3 h)^45^ was used. Total minutes of moderate‐to‐vigorous physical activity was calculated by summing the total number of vigorous physical activity minutes (minutes per day × number of days) and total number of moderate physical activity minutes (minutes per day × number of days) in the past week.

#### 2.2.4 Screen time

Participants reported the average number of minutes they spent per day watching television separately on weekdays and weekend days. Minutes of computer activity for leisure were also reported. Total minutes of leisure screen time^46^ was calculated by summing hours spent watching television per week [(weekday hours × 5 days) + (weekend hours × 2 days)] and hours of leisure computer activity per week [(weekday hours × 5 days) + (weekend hours × 2 days)].

### 2.3 Statistical analysis

All analyses were performed in SPSS Statistics Version 24. A total of 0.41% of the data were missing. The initial subsample included 663 young adults, of which 24 (3.6%) had a missing or implausible physical activity value, nine (1.4%) were missing a screen time value, two had skipped the body‐related shame and guilt questions (0.3%) and six (0.9%) were missing ethnicity. As per the International Physical Activity Questionnaire scoring instructions,^45^ individuals with implausible or missing values were excluded. Participants with missing or impossible screen time, ethnicity, shame or guilt values were also excluded. Assumptions for regression analyses were assessed and managed following established guidelines.^47^

Descriptive statistics and Spearman rho's correlations were calculated for and between each variable. A chi‐square analysis compared the frequency of weight status by sex. The PROCESS macro,^48^ which estimates mediation with 10 000 bootstrapped re‐samples and bias‐corrected confidence intervals, was used. A mediating (indirect) effect was considered significant if the 95% confidence interval did not include zero.^48^, ^49^ Importantly, the absence of a direct effect does not negate the presence of an indirect effect.^49^ Proposed mediators were simultaneously entered into a single model, with separate models estimated for physical activity and screen time. BMI, sex^50^ and ethnicity (Caucasian, non‐Caucasian)^20^ were entered as covariates to isolate the associations among weight perception, physical activity and screen time. Given the dichotomous nature of the independent variable, partially standardized effect sizes and associated confidence intervals are also reported.^48^

## 3 RESULTS

Descriptive statistics are presented in Table 1, and correlations are presented in Table 2. Seventy‐one per cent of females and 55% of males were within a normal weight range, and 29% of females and 45% of males had overweight or obesity. Most normal weight males (87%) and females (75%) perceived themselves as just about right. Sixty per cent of males and 89% of females who had overweight or obesity perceived themselves as too heavy. More females than males perceived themselves as too heavy when their BMI was normal [*χ* ^2^(1) = 8.20, *p* < .01], and more males than females perceived themselves as about right when they had overweight or obesity [*χ* ^2^(1) = 23.79, *p* < .001]. Compared with males, females had a lower BMI, experienced more shame and guilt and engaged in less physical activity and screen time.

**TABLE 1:** Demographic information

	About right (*n* = 375)	Too heavy (*n* = 243)
Age (years) M (SD)	24.02 (0.67)	24.07 (0.59)
Body mass index (kg/m^2^) M (SD)	22.42 (2.29)	27.19 (4.49)
Female, %	54	64
Single, %	70	72
White, %	84	82
Shame M (SD)	0.41 (0.54)	1.23 (0.95)
Guilt M (SD)	0.78 (0.76)	1.76 (1.04)

**TABLE 2:** Descriptive statistics and bivariate correlations between weight, weight perceptions, body‐related self‐conscious emotions and lifestyle behaviours (Spearman's rho)

	1	2	3	4	5	6	7	8
1. Weight perception	‐							
2. Sex	.093^*^	‐						
3. Ethnicity	.033	.018	‐					
4. BMI	.57^**^	−.23^**^	.026	‐				
5. Shame	.48^**^	.36^**^	−.009	.26^**^	‐			
6. Guilt	.46^**^	.32^**^	.015	.28^**^	.74^**^	‐		
7. MVPA (min/week)	−.068	−.22^**^	−.13^**^	.11^**^	−.15^**^	−.016	‐	
8. ST (min/week)	.13^**^	−.18^**^	.11^*^	.11^**^	.10^*^	.018	−.14^**^	‐

Abbreviations: BMI, body mass index; MVPA, minutes of moderate‐to‐vigorous physical activity; ST, minutes spent sedentary watching television and using the computer for leisure.

* *p* < .05.

** *p* < .01.

Shame mediated the relationships among weight perception, physical activity and screen time (95% CI = −89.60, −28.26 and 57.05, 227.15, respectively), such that perceiving oneself as too heavy was associated with higher levels of shame, which was associated with lower physical activity and higher screen time. Guilt mediated the relationship between weight perception and physical activity (95% CI = 9.20, 82.51), such that perceiving oneself as too heavy was associated with higher levels of guilt, which was associated with higher physical activity. No indirect effect between weight perception and screen time was observed (95% CI = −170.76, 13.91) (Table 3).

**TABLE 3:** Estimated effects of weight perception on self‐conscious emotions, physical activity and screen time

IV	DV	M	*a*	*b*	*c′*	*a* × *b* Point estimate [95% CI]	*a × b*_*ps*_ Point estimate [95% CI]
Weight perception	MVPA: *R* ^2^ = 9.0%	Shame	.59 (.07)^**^	−97.41 (26.75)^**^	−41.36 (37.67)	−57.40 [−89.60, −28.26]^*^	−0.16 [−0.25, −0.080]
		Guilt	.78 (.09)^**^	58.02 (21.71)^*^		44.52 [9.20, 82.51]^*^	0.13 [0.026, 0.23]
	ST: *R* ^2^ = 8.0%	Shame	.59 (.07)^**^	231.22 (69.32)^**^	147.48 (97.63)	136.25 [57.05, 227.15]^*^	.15 [0.062, 0.25]
		Guilt	.78 (.09)^**^	−97.36 (56.28)		−74.70 [−170.76, 13.91]	−0.082 [−0.19, 0.015]

Abbreviations: CI, confidence interval; MVPA, moderate‐to‐vigorous physical activity; *ps*, partially standardized effect; *R* ^2^, adjusted *R* ^2^; ST, screen time.

* *p* < .05.

** *p* < .001.

## 4 DISCUSSION

This study examined the relationships among weight perception, body‐related self‐conscious emotions, physical activity and screen time in young adults. The hypothesis that shame and guilt mediate the associations among weight perceptions, physical activity and screen time was partially supported. Perceiving oneself as too heavy was associated with higher levels of shame, which was in turn was associated with lower physical activity and higher screen time. Perceiving oneself as too heavy was also associated with higher levels of guilt, which was associated with higher physical activity. These findings advance our understanding of the unique contributions of weight perception on lifestyle behaviours that influence weight management and health in young adults.

Perceiving weight as about right relative to too heavy was associated with less shame and guilt, even after controlling for weight status. These findings challenge assumptions that all individuals who have overweight or obesity experience heightened shame and guilt due to their weight status, and highlight the importance of perceptions in the manifestation of these emotions. Similar to obesity‐related hypotheses,^34^, ^35^ elevated shame and guilt associated with perceiving oneself as too heavy may be due to the pervasive stigma and bias associated with obesity. Individuals with obesity are stereotyped as lazy, unmotivated and lacking self‐discipline and are often blamed for their weight status.^51^, ^52^ It is not surprising that perceiving oneself as discrepant from the normative ideal due to global flaws would elicit shame.^36^, ^53^ In regard to guilt, the bombardment of exercise, diet and weight loss promotion from mainstream and social media (i.e., ‘diet culture’)^54^, ^55^ may contribute to the belief among individuals who perceive themselves as too heavy that they are not doing enough to achieve a lower weight. Reducing intrinsic blame associated with weight by educating individuals on the role of other, uncontrollable factors that influence weight (i.e., built environment, genetics, socio‐economic status and food availability)^56^ and disentangling media messaging and the realities of weight management, may alleviate shame and guilt experienced by those who perceive themselves as too heavy.^57^

While increasing evidence highlights that individuals who perceive themselves as too heavy engage in less physical activity and more sedentary behaviours (such as screen time),^21^, ^28^, ^29^, ^30^ these findings offer unique insights into the potential mechanistic role of self‐conscious emotions. Consistent with theoretical tenants^36^ and empirical evidence,^39^, ^41^ higher levels of shame were associated with less physical activity. A novel finding was that higher levels of shame were also associated with more screen time. Shame is associated with avoidance of social contexts,^58^ suggesting that people with body‐related shame may avoid social situations in which they perceive their body as being evaluated or on display.^39^ This avoidance may manifest as higher levels of screen time. Interventions that target body‐related shame may be important to promote physical activity and reduce screen time among young adults who perceive themselves as too heavy.

Guilt was related to higher levels of physical activity but not lower levels of screen time. Guilt is theorized to increase levels of physical activity, because activity may serve as a compensatory strategy to ‘fix’ poor weight control efforts such as overeating.^38^, ^39^ A novel finding was that levels of guilt were not associated with screen time. Importantly, this is not contradictory to the finding that high levels of guilt are associated with higher physical activity. Sedentary time and physical activity are not inverse behaviours, such that individuals can accrue high levels of both physical activity and sedentary time.^59^ This study is one of the first examinations of the relationship between body‐related guilt and a sedentary behaviour such as screen time, and more work investigating whether limiting sedentary time may be perceived as a reparative weight‐related behaviour is necessary.

Although shame and guilt are often highly correlated, these emotions tend to have opposite effects on behaviour (i.e., shame leads to avoidance of physical activity, and guilt leads to reparative physical activity).^36^, ^39^ Such patterns were evident in the present sample. The potential for these contradictory emotions to ‘cancel out’ the effects of a too heavy weight perception on behaviour may in part explain why weight perceptions have also had null associations with physical activity behaviour.^26^ However, the Process Model of Self‐Conscious Emotions suggests that multiple self‐conscious emotions can be elicited in response to a single event.^36^ The variance in the temporal nature of these emotions (i.e., is shame an antecedent to guilt or are they simultaneously elicited) is also not well understood. Therefore, capturing acute fluctuations in self‐conscious emotions using ecological momentary assessment^60^ may better elucidate how state shame and guilt differentially influence behaviour within an individual.

The guilt and increased physical activity associated with perceiving oneself as too heavy may be maladaptive. Body‐related guilt has been positively associated with extrinsic physical activity regulations, which are associated with less sustainable physical activity behaviours.^36^, ^41^, ^61^ Individuals who exercise for appearance and weight‐related reasons also endorse higher levels of body dissatisfaction, depression, anxiety and disordered eating symptoms than those who exercise for health and wellness.^62^, ^63^ Guilt is also identified as a subcomponent of rule‐driven behaviours that predict compulsive exercise.^64^ Therefore, higher perceptions of guilt linked to higher physical activity levels may have negative implications on overall physical and psychological health.

Our findings suggest that perceiving oneself as about right may be protective against lower physical activity levels and greater screen time in young adults who have overweight or obesity. This evidence contradicts the pervasive ‘ignorance is damaging’ attitude, which argues that being aware of one's overweight or obesity status is critical for engagement in weight control practices.^65^ Such views are evident in legislated school obesity screening programmes and health care practitioner behaviours that strive to identify and notify individuals whose BMIs are considered overweight.^66^, ^67^ The current findings contribute to growing evidence that this awareness may be a less critical component of weight management than initially anticipated. Adolescents informed by their physician about their weight status were more likely to report a desire to weigh less, and eat significantly less, and yet showed no difference in physical activity behaviour.^68^ In contrast, adolescents with overweight and obesity who misperceive their weight status as normal experienced less BMI gain over 12 years than those who correctly perceived their weight status,^69^ while perceiving oneself as overweight (regardless of accuracy) predicts long‐term weight gain.^22^ Moving forward, it is essential to consider optimal health care practices and policies that encourage healthy eating and exercise while being mindful of the possible negative implications of inducing overweight or obesity perceptions. It is also important to consider sex differences in these perceptions. Consistent with previous evidence, females were more likely to endorse a too heavy perception than males,^21^ as well as experience higher levels of body‐related shame and guilt.^50^ Tailoring interventional programmes and practices to sex‐specific sociocultural pressures that may contribute to perceptual and affective body image (e.g., the female thin‐ideal versus the male mesomorphic ideal)^70^, ^71^ may be an important consideration moving forward.

Limitations of this study include that physical activity and screen time behaviour were measured in self‐reports, which can lead to less accurate estimates relative to a more objective measure like accelerometery.^72^ In addition, because screen time is just one component of sedentary behaviour, a more comprehensive measure should be used in future research. Further, self‐conscious emotions were assessed as they relate to weight, yet body‐related emotions are also explored as contingent on appearance and function/fitness.^42^ Because individuals with obesity often endorse physical challenges in addition to shame during physical activity,^73^ it is important to further examine how different facets of self‐conscious emotions may uniquely impact physical activity and sedentary behaviours. Although consistent with theoretical tenants and empirical evidence,^36^, ^41^ the cross‐sectional nature of the data precludes the ability to make causal inferences regarding the directionality of these relationships. Lastly, positive body‐related self‐conscious emotions (i.e., hubristic and authentic pride) that have been linked to physical activity behaviour were not assessed.^74^, ^75^ Therefore, future longitudinal research that incorporates both positive and negative self‐conscious emotions may provide more well‐rounded insight into how weight perception is associated with physical activity and sedentary behaviour.

By considering the role of weight perception, this study offers a novel approach in investigating the associations among self‐conscious emotions, physical activity and the sedentary behaviour of screen time. Regardless of accuracy, perceiving oneself as too heavy is indirectly associated with physical activity and screen time, and this relationship is mediated by shame and guilt. This research offers preliminary insight into how weight perception may impact engagement in physical activity and screen time and suggests that targeting self‐conscious emotions could be beneficial for promoting health behaviours in young adults with overweight and obesity.

## CONFLICTS OF INTEREST STATEMENT

The authors declare that there is no conflict of interest.

## FUNDING

This work was supported by the Canadian Cancer Society (Grants 010271, 017435 and 704031). C.M.S. holds a Canada Research Chair in Physical Activity and Mental Health, and J.O.L. holds a Canada Research Chair in the Early Determinants of Adult Chronic Disease. Funding sources were not involved in the study design, data collection, analysis or interpretation or the preparation of the manuscript for publication.
